# PKHB1 Tumor Cell Lysate Induces Antitumor Immune System Stimulation and Tumor Regression in Syngeneic Mice with Tumoral T Lymphoblasts

**DOI:** 10.1155/2019/9852361

**Published:** 2019-06-04

**Authors:** Ana Carolina Martínez-Torres, Kenny Misael Calvillo-Rodríguez, Ashanti Concepción Uscanga-Palomeque, Luis Gómez-Morales, Rodolfo Mendoza-Reveles, Diana Caballero-Hernández, Philippe Karoyan, Cristina Rodríguez-Padilla

**Affiliations:** ^1^Universidad Autónoma de Nuevo León, Facultad de Ciencias Biologicas, Laboratorio de Inmunología y Virología, Mexico; ^2^Sorbonne Université, École Normale Supérieure, PSL University, CNRS, Laboratoire des Biomolécules, 75005 Paris, France; ^3^Kayvisa, AG, Industriestrasse 44, 6300 Zug, Switzerland; ^4^Kaybiotix, GmbH, Zugerstrasse 32, 6340 Baar, Switzerland

## Abstract

Acute lymphocytic leukemia (ALL) is the most common pediatric cancer. Currently, treatment options for patients with relapsed and refractory ALL mostly rely on immunotherapies. However, hematological cancers are commonly associated with a low immunogenicity and immune tolerance, which may contribute to leukemia relapse and the difficulties associated with the development of effective immunotherapies against this disease. We recently demonstrated that PKHB1, a TSP1-derived CD47 agonist peptide, induces immunogenic cell death (ICD) in T cell ALL (T-ALL). Cell death induced by PKHB1 on T-ALL cell lines and their homologous murine, L5178Y-R (T-murine tumor lymphoblast cell line), induced damage-associated molecular patterns (DAMPs) exposure and release. Additionally, a prophylactic vaccination with PKHB1-treated L5178Y-R cells prevented tumor establishment* in vivo *in all the cases. Due to the immunogenic potential of PKHB1-treated cells, in this study we assessed their ability to induce antitumor immune responses* ex vivo* and* in vivo *in an established tumor. We first confirmed the selectivity of cell death induced by PKBH1 in tumor L5178Y-R cells and observed that calreticulin exposure increased when cell death increased. Then, we found that the tumor cell lysate (TCL) obtained from PKHB1-treated L5178YR tumor cells (PKHB1-TCL) was able to induce,* ex vivo*, dendritic cells maturation, cytokine production, and T cell antitumor responses. Finally, our results show that* in vivo*, PKHB1-TCL treatment induces tumor regression in syngeneic mice transplanted with L5178Y-R cells, increasing their overall survival and protecting them from further tumor establishment after tumor rechallenge. Altogether our results highlight the immunogenicity of the cell death induced by PKHB1 activation of CD47 as a potential therapeutic tool to overcome the low immunogenicity and immune tolerance in T-ALL.

## 1. Introduction

T cell acute lymphoblastic leukemia (T-ALL) is a hematological malignancy that affects mostly pediatric patients, as they account for 80% of the cases [1, 2]. It represents the second most common acute leukemia in adults, with a 5-year survival rate of about 30-50% [3–5] with a high risk of relapse [6]. The use of nelarabine for relapsed and refractory T-ALL only results in responses in a substantial minority of patients [7]. Among other treatments, allogeneic hematopoietic cell transplantation (HCT) is proposed in patients with high-risk or relapsed/refractory disease, and *γ*-secretase inhibitors for patients with NOTCH1 mutations are currently in clinical trials. Multiagent chemotherapy is proposed for older and unfit patients. However, T-ALL treatments have lagged behind those proposed for B-cell ALL, and the development of new therapeutic approaches against this aggressive malignancy remains a challenge. Since the T-ALL high risk of relapse has been attributed to its low immunogenicity and immune tolerance [6], the immune system stimulation able to induce immunological memory against tumor cells appears as a challenging but promising goal.

With this aim, whole tumor cell lysates (TCLs) have been shown to be able to prompt antitumor immune responses in preclinical murine models for glioblastoma, breast, and ovarian cancer and in clinical trials for melanoma, prostate, and ovarian cancer [8]. These immune responses are correlated with damage-associated molecular patterns (DAMPs) induction and the availability of the tumor neoantigens, both of which are promoted in accordance with the specific cell death inductor [9]. DAMPs interact with dendritic cells' (DCs') receptors (CD91, Toll-like receptor 4, purinergic receptors, among others), promoting their maturation and increasing antitumor activity [10]. Thus, TCL can be used to induce an immunogenic response from DCs against multiple tumor antigens, triggering a polyclonal tumor-specific T cell response [11].

Cancer treatment with DCs pulsed with tumor antigens has proved effective antitumor responses in mesothelioma, glioma, and breast cancer [12–14]. However, the use of TCL as therapeutic vaccines has been also shown to be a useful strategy to elicit antitumor immune responses, while overcoming immunosuppressive mechanisms of the tumor microenvironment [8]. TCLs hold more promises as cancer vaccines than individual tumor-associated antigens (TAAs) because they can elicit immune responses to multiple TAAs [15]. However, the availability and types of neoantigens, the amount of DAMPs released, and the overall immunogenicity of the TCL strongly rely on the cell death inductor [9]. Thus, it is important to find effective cell death inductors that are able to provide an immunogenic TCL able to induce antitumor immune responses.

CD47 activation through coated [16–18] or soluble anti-CD47 antibodies [19, 20], or immobilized [16] or soluble peptides derived from the C-terminal domain of thrombospondin-1 [21, 22], is an effective way to induce cell death in different types of cancerous cells, even in cells coming from patients that are resistant to chemotherapy [21, 22]. Thus, a TCL obtained through CD47 activation might help to understand the implications of CD47-mediated cell death in the activation of antitumor immune responses. Recently, we have shown that treatment of T-ALL cells with the CD47-agonist peptide, PKHB1, induced immunogenic cell death (ICD) [23]. ICD was induced by PKHB1 in T-ALL cells, while it spares CD19 and CD3 lymphocytes [21], human and murine PBMCs, CD4 and CD8 T cells, and cells from murine lymphoid organs [23]. We found that PKHB1-treatment induces the exposure and release of several DAMPs (calreticulin (CRT), HSP70, HSP90, ATP, and HMGB1) in human T-ALL cell lines (CEM, MOLT-4) and their murine counterpart (L5178Y-R cells) [23].* In vivo,* prophylactic vaccination experiments with PKHB1-treated cells prevented tumor establishment in immunocompetent BALB/c mice [23]. These results demonstrated that CD47 activation by PKHB1 was able to induce DAMPs release and provide neoantigens able to elicit an antitumor immune response that prevented tumor establishment. However, the therapeutic potential of this type of ICD was not studied.

In the present work we focused on determining whether the induction of ICD by PKHB1 has a therapeutic potential. Due to the immunogenicity of PKHB1-treated cells, we used the TCL obtained from PKHB1-treated L5178Y-R tumor cells (PKHB1-TCL) and focused on determining their ability to induce antitumor immune responses* ex vivo* and* in vivo *in an established L5178Y-R tumor developed in syngeneic BALB/c mice.

## 2. Material and Methods

### 2.1. T Cells and Dendritic Cells (DCs)

This study was approved by the Animal Ethical Committee (CEIBA), of the School of Biological Sciences Number: 01/2015. All experiments were conducted according to Mexican regulation NOM-062-ZOO-1999.

The blood from sacrificed BALB/c mice was obtained by cardiac puncture. Peripheral blood mononuclear cells (PBMCs) isolation was performed by density gradient centrifugation using Ficoll-Hypaque-1119 (Sigma-Aldrich, St Louis, MO, USA). Murine CD3+ cells were isolated from total PBMCs by positive selection using magnetic-activated cell sorting (MACS) microbead technology with anti-CD3*ε*-biotin and anti-biotin microbeads (Miltenyi Biotech; >98% purity and >98% viability), as stated by manufacturer's instructions.

To obtain bone marrow-derived dendritic cells (DCs), after sacrifice, mice bone marrow was removed from femur and tibia of female BALB/c mice by flushing into RPMI-1640. Eluted cells were cultured for 5 days with 20 ng/mL of IL-4 and GM-CSF (R&D Systems, Minneapolis, MN, USA) until approximately 70% of the cells were CD11c+.

### 2.2. Cell Culture

L5178Y-R cell line (murine cancerous T lymphoblasts) was obtained from the ATCC. L5178Y-R, primary murine CD3+, and DCs were maintained in RPMI-1640 medium supplemented with 10% of fetal bovine serum, 2 mM L-glutamine, and 100U/mL penicillin-streptomycin (GIBCO by Life Technologies, Grand Island, NY, USA), and incubated at 37°C in a controlled humidified atmosphere with 5% CO_2_. Cell count was performed using trypan blue (0.4% Sigma-Aldrich), a Neubauer chamber, and an optic microscope (Zeiss Primo Star) as proposed by the ATCC's standard protocols.

### 2.3. Cell Death Analysis

Annexin-V-allophycocyanin (Ann-V-APC 0.1*μ*g/ml; BD Pharmingen, San Jose, CA, USA) and propidium iodide (PI, 0.5*μ*g/ml Sigma-Aldrich) were used to assess phosphatidylserine exposure, cell death, and cell viability quantification, respectively, in a BD Accuri C6 flow cytometer (BD Biosciences, Franklin Lakes, NJ, USA) (total population: 10,000 cells). Data was analyzed using FlowJo software (LLC, Ashland, OR, USA). 1X10^6^ cells/mL were seeded and left untreated or treated for 2 h with 150 *μ*M or 300 *μ*M of PKHB1 (KRFY**VV**MWKK) or 150 *μ*M of the control peptide 4NGG (KRFY**GG**MWKK) (as indicated) in serum-free media.

For cell death inhibitions we used the calcium chelator BAPTA (5mM), the antioxidant N-Acetyl Cysteine (NAC, 5 mM), the pan-caspase inhibitors Q-VD-OPH (QVD, 10 *μ*M) and Z-VAD-FMK (Z-VAD, 50*μ*M), the autophagic inhibitor Spautin-1 (SP-1, 15 *μ*M), and the necroptotic inhibitor Necrostatin-1 (Nec-1, 50 *μ*M). We pretreated the cells 30 minutes with the inhibitor before the treatment with PKHB1 (150 *μ*M).

### 2.4. Calreticulin Exposure

L5178Y-R cells were plated (1x10^6^ cells/mL), left untreated or treated with 300 *μ*M of 4NGG or 150 *μ*M or 300 *μ*M of PKHB1, and incubated for 2 h. Cells were harvested, washed, and stained with Calreticulin-Phycoerythrin (Calreticulin-PE, FMC-75; Enzo Life Science, Farmingdale, NY, USA) antibody (1:1000) in FACS buffer. After 1 h in darkness at room temperature (RT), cells were washed and resuspended in 100 *μ*L FACS buffer (PBS 1x and 2% of fetal calf serum) to be assessed by flow cytometry in a BD Accuri C6 flow cytometer (BD Biosciences) (total population: 10,000 cells). Data was analyzed using FlowJo software.

### 2.5. DCs Markers

DCs (1 × 10^6^ cells/mL) were stained in 100 *μ*L of FACS buffer with the indicated antibodies at RT for 30 minutes and then washed twice with PBS. The cell surface markers were evaluated by flow cytometry with the fluorescent label-conjugated antibodies, anti-CD11c-Alexa-fluor 488 (R&D Systems), anti-CD80-FITC, and anti-CD86-APC, from BD Biosciences (San Jose, CA, USA).

### 2.6. Cocultures

DCs-PKHB1 tumor cell lysate: DCs were resuspended in fresh medium at a concentration of 1 × 10^6^ cells/mL. DCs were left untreated (control), or PKHB1-treated tumor cells were added at a concentration of 3 × 10^6^ cells/mL to give a range of 1:3 DCs to PKHB1-treated tumor cells ratios. Coculture was left for 24 hours. Then the supernatant was removed, and the well was washed twice with PBS before doing the next coculture (with the addition of T-lymphocytes).

DCs-T-lymphocytes: Control DCs or DCs previously cocultured with PKHB1-TCL were maintained in fresh medium at a concentration of 1 × 10^6^ cells/mL. Then, allogeneic BALB/c mCD3+ cells were added to each well at 3 × 10^6^ cells/mL to give a range of 1:3 DC to CD3+ cells ratios. Coculture was left for 96 hours. Then, the lymphocytes were collected (by obtaining the supernatant), washed with PBS, and resuspended in fresh medium at a concentration of 5x10^6^ cells/mL to be used in the next coculture (T- lymphocytes with cancer cells).

T-Lymphocytes-L5178Y-R cells: viable L5178Y-R cells were plated at a concentration of 1 × 10^5^ cells/mL. Then, unprimed (previously cocultured with control DCs) or primed (previously cocultured with DCs-PKHB1-TCL) allogeneic BALB/c mCD3+ cells were added to each well at 5 × 10^5^ cells/mL to give a range of 1:5 tumor to effector ratios. Coculture was left for 24 hours, before cytokine or calcein assessment.

### 2.7. Cytokine Release Assay

The supernatants from the indicated cultures were collected for IL-2, IL-4, IL5, and TNF*α* assessment (BD CBA Mouse Th1/Th2 Cytokine Kit, San Jose, CA, USA) by flow cytometry following manufacturer's instructions. IFN*γ* was assessed using an ELISA kit (Sigma-Aldrich) and using the Synergy HTTM (BioTek Instruments, Inc., Winooski, VT, USA) plate reader at 570 nm wavelength, following manufacturer's instructions.

### 2.8. Calcein Assay

L5178Y-R cells (1 × 10^6^ cells/mL) were stained with (0.1*μ*L/mL) of Calcein-AM from BD Biosciences (San Jose, CA) for 30 minutes and washed twice (PBS sterile). After this, T cells previously primed with DCs pulsed with PKHB1-TCL or with unpulsed DC were added in a 1:5 ratio. The L5178Y-R-T-lymphocytes' coculture was incubated at 37°C and 5% CO2 for 24 h. Finally, L5178Y-R-calcein negative cells were assessed in a BD Accuri C6 flow cytometer (BD Biosciences) (total population: 10,000 cells). Data was then analyzed using FlowJo software.

### 2.9. In Vivo Model

Six-to-eight-week-old BALB/c female mice were maintained in controlled environmental conditions (25°C and 12 h light/dark cycle) and were supplied with rodent food (LabDiet, St. Louis, MO, USA) and water* ad libitum*.

Tumor was established by subcutaneous injections of 2x10^6^ L5178Y-R cells in 100 *μ*L PBS, in the left hind. Tumor volume and mice weight were measured three times a week using a caliper (Digimatic Caliper Mitutoyo Corporation, Japan) and a digital scale (American Weigh Scale-600-BLK, USA), respectively. Tumor volume was determined with the following formula: tumor volume (mm^3^) = 4*π*/3*∗*A(length)*∗*B(width)*∗*C(height). When the tumor reached 100 mm^3^, the first therapeutic vaccine of PKHB1-tumor cell lysate (PKHB1-TCL) was applied as follows:

L5178Y-R cells (5x10^6^) were treated* in vitro* with 300 *μ*M PKHB1 for 2 h (CC_100_) in serum-free RPMI medium. Cell death was confirmed as previously indicated. Treated cells were inoculated subcutaneously in 100 *μ*l serum-free media, in the right hind, twice a week. Controls were treated with 100 *μ*l serum-free media.

For long memory assessment, we used six naïve mice (control) and six mice in complete remission after PKHB1-TCL treatment (tumor free >60 days). Both groups were injected with 2x10^6^ living L5178Y-R cells in 100 *μ*L PBS, in the left hind. The latter group was named PKHB1-TCL-Rechallenge. We then assessed tumor volume and survival, as described previously.

### 2.10. Statistical Analysis

Mice were randomly assigned to different groups for all* in vivo* studies. At least three independent experiments were repeated three independent times. Mann-Whitney tests and two-tailed unpaired Student's* t*-tests were performed using GraphPad Prism Software (San Diego CA, USA) and presented as mean values ±SD. The *p* values were considered significant as follows:* p*<0.05,* p*<0.01, and* p*<0.001.

## 3. Results and Discussion

### 3.1. Calreticulin Exposure Correlates with Cell Death Induced by PKHB1

ICD is characterized by DAMPs exposure or release, and anticancer immune memory generation [24]. CRT exposure has been continuously reported as one of the principal DAMPs necessary for the correct maturation of DCs and antigen presentation [25, 26]. The activation of CD47 by PKHB1 induces CRT exposure in CLL cells [21]. Additionally, we recently reported that PKHB1 induces immunogenic cell death with DAMPs release (CRT, HMGB1, HSP79, HSP90, and ATP) and CRT exposure in T -ALL human cell lines and their murine counterpart, the L5178Y-R cell line (a murine T cell lymphoblastic tumor cell line) [23]. However, correlation between CRT exposure and cell death induced by CD47 was not established; for that purpose, here we assessed this feature using the L5178Y-R cell line.

First, we assessed cell death induced by the control peptide 4NGG, which does not bind to CD47 [21], and cell death induced by different concentrations of PKHB1. We found that 4NGG (300 *μ*M) was not able to induce cell death in L5178Y-R cells, while PKHB1 induced a concentration-dependent cell death, reaching CC_50_ (cytotoxic concentration for 50% of the cells) at 150 *μ*M and CC_100_ (cytotoxic concentration for 100% of the cells) at 300 *μ*M.

**Figure 1:**
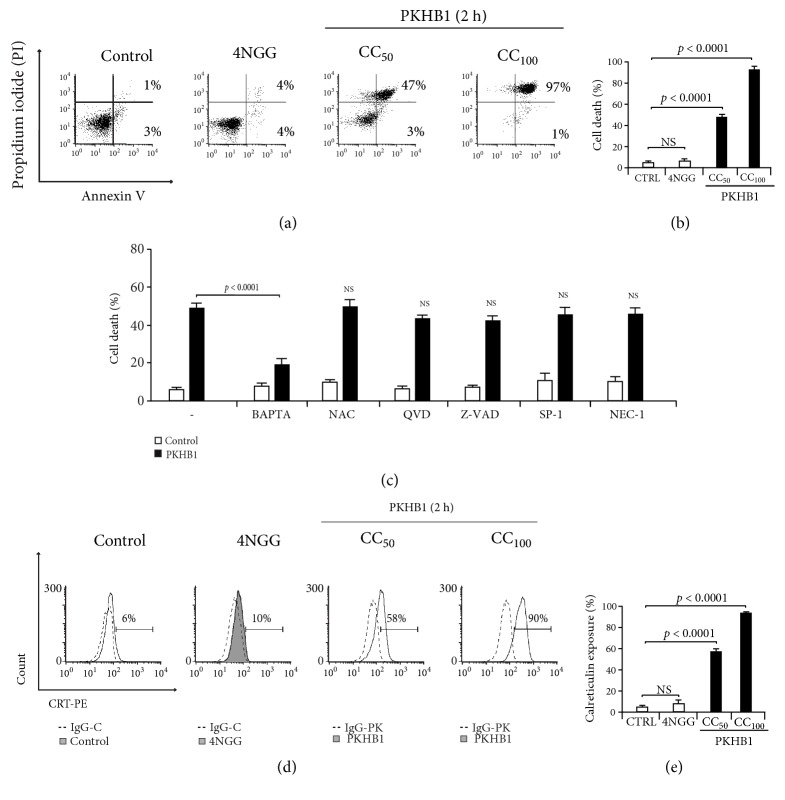
*PKHB1 induces cell death and calreticulin exposure in L5178Y-R cell line.* (a) Cell death was measured by Annexin-V-allophycocyanin (Annexin-V-APC) and propidium iodide (PI) staining and graphed. Dot plots of L5178Y-R cells untreated (control) and treated with control peptide 4NGG (300 *μ*M) or CC_50_ (150 *μ*M) and CC_100_ (300 *μ*M) of PKHB1 for 2 h. (b) Graph represents the means (±SD) of triplicates of three independent experiments obtained as in (a). (c) Cell death induced by PKHB1 was assessed as in (a) with cells left without pretreatment (control) or pretreated (30 minutes) with BAPTA, N-Acetyl Cystein (NAC), Q-VD-OPH (QVD), Z-VAD-FMK (Z-VAD), Spautin-1 (SP-1), or Necrostatin-1 (Nec-1). (d) Calreticulin exposure induced by 4NGG (300 *μ*M) and PKHB1 (CC_50_ and CC_100_, 2 h) was measured using FACS in L5178Y-R cell line, and representative histograms are shown. (e) Graph represents the means (±SD) of triplicates of three independent experiments obtained as in (c).

Next, to evaluate the characteristics of the cell death induced by PKHB1, we used several cell death inhibitors. We have previously demonstrated that cell death induced through CD47 activation by PKHB1 is a fast and atypical caspase-independent and calcium-dependent mechanism [21, 23]. Thus, we assessed cell death using the calcium chelator BAPTA, as positive control of cell death inhibition by PKHB1, and the antioxidant NAC (N-Acetyl Cysteine) which inhibits several cell death modalities that involve ROS production [27]; as apoptotic pan-caspase inhibitors we used Q-VD-OPH [28] and Z-VAD-FMK (which also inhibits pyroptosis, [29]); we also used the autophagic inhibitor Spautin-1 [30] and the necroptotic inhibitor Necrostatin-1 [31]. In Figure 1(c) we can observe that that only the calcium chelator, BAPTA, was able to inhibit PKHB1-cell death. Effectively cell death induced by CD47 activation seems to be mostly cytoplasmic and mediated by calcium augmentation [21, 23], and due to the velocity of the process it seems to be a different mechanism of cell death from the commonly described to date.

Using 300 *μ*M of PKHB1 for two hours induced 97% of cell death (defined as CC_100_), and calreticulin exposure was observed for 90% of the cells. The PKHB1-tumor cell lysate of L5178Y-R cells (PKHB1-TCL) was generated with this CC_100_.  Figure 1 describes the PKHB1-induced CRT exposure in a PKHB1-concentration and cell death-dependent ways: indeed, the increasing number of Ann-V-APC/PI positive cells with increasing concentration of PKHB1 (Figures 1(a) and 1(b)) is correlated with an increasing CRT exposure (Figures 1(d) and 1(e)).

Calreticulin exposure and cell death have been shown to be correlated when using various agents inducing ICD [26], such as IMMUNEPOTENT CRP [32] and shikonin [33]; however in some cases, CRT has been reported to be exposed premortem [26]. We recently found that the CC_100_ was necessary for the highest release of HMGB1, HSP70, and HSP90 in L5178Y-R cells [23]. This observation led us hypothesize that since the PKHB1-TCL is rich in DAMPs, it might induce DCs maturation and antigen presentation to T cells promoting antitumor responses.

### 3.2. PKHB1-TCL Induces Maturation of Bone Marrow-Derived DCs

**Figure 2:**
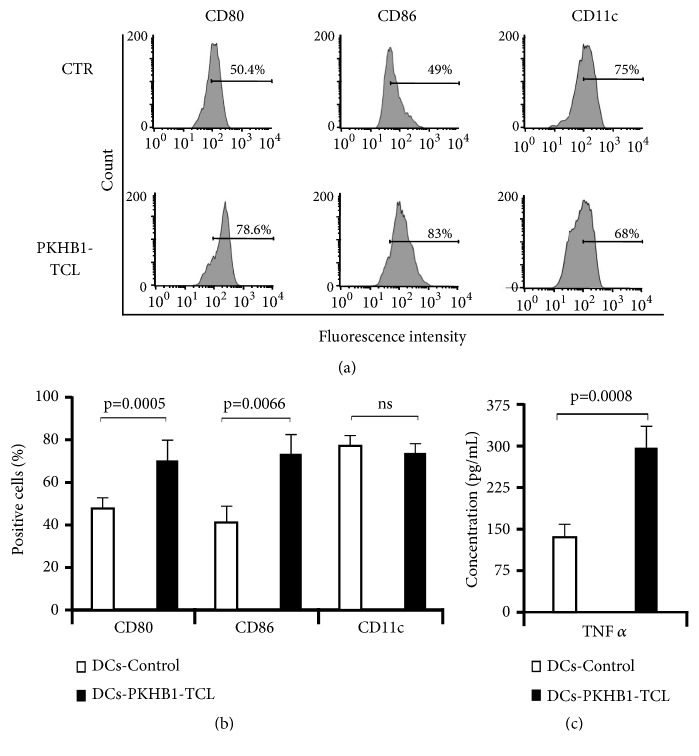
*PKHB1-tumor cell lysate induces the maturation of bone marrow-derived dendritic cells.* (a) Bone marrow-derived murine DCs were left only with medium (control) or pulsed during 24 h with a PKHB1-TCL. DCs were then stained to assess cell surface markers (CD11c, CD80, or CD86) by FACS, and representative histograms are shown. (b) DCs were treated as in (a) and the means obtained by FACS were graphed. (c) DCs were treated as in (a) and the supernatants were collected to quantify TNF*α* release, by FACS. Graphs represent the means (±SD) of triplicates of at least three independent experiments.

To determine if PKHB1-TCL was able to induce the maturation of DCs, bone marrow-derived murine DCs were left untreated (control) or pulsed for 24 h with the previously obtained PKHB1-TCL. We assessed cytokine production by PKHB1-TCL, but we did not find a significant release of TNF*α*, IFN*γ*, IL-5, IL-4, or IL-2 (Supplementary Table 1). After coculture, DCs were washed twice with PBS to remove any background noise given by the PKHB1-TCL. DCs cocultured with PKHB1-TCL show morphological changes (data not shown) and a significant increase in the expression of costimulatory molecules (CD80 and CD86) passing from 50% to 78%, while maintaining the expression of the DCs marker CD11c (Figures 2(a) and 2(b)). Furthermore, only DCs-PKHB1-TCLs show a significant increase in TNF*α* release in comparison with unstimulated DCs (Figure 2(c)).

Several types of TCL are able to induce DCs maturation at different degrees [8]; however most of them use LPS [33] or other adjuvants such as phytoextracts [34] and bacterial ghosts [35] in combination with the TCL. Our results show that PKHB1-induced cell death is able to promote DCs maturation and secretion of TNF*α*, even in the absence of other immune-stimulants. The mature DC phenotype was characterized by the expression of the endocytic receptor CD11c [36], CD80, and CD86 [37], which increased significantly (p=0.0005 and p=0.0066, respectively) in DCs cocultured with PKHB1-TCL. We can observe a slight nonsignificant decrease in the expression of CD11c (Figures 2(a) and 2(b)); this differentiation marker can be downregulated by dendritic cells after their activation by TLR agonists [38]. The secretion of TNF*α* has been associated with a mature phenotype, as it acts as an autocrine maturation factor for DCs [37]. Several TCLs are able to induce its secretion at several degrees, ranging from 20 pg/mL to 250 pg/mL [39, 40]. Here we found that DCs pulsed with PKHB1-TCL induced the secretion of TNF*α* at a 270 pg/mL concentration, indicating the efficient maturation of DCs by PKHB1-TCL.

### 3.3. PKHB1-TCL Induces an Antitumor T Cell Response

Once we determined that PKHB1-TCL was able to induce DCs maturation, we assessed if the pulsed DCs (DCs-PKHB1-TCL) were able to prime T cells. First, CD3+ cells were cocultured for four days with pulsed or unpulsed DCs, and we assessed TNF*α*, IFN*γ*, IL-5, IL-4, and IL-2 cytokine release.  Table 1 shows that coculture of pulsed DCs with primary T-lymphocytes induces the release of TNF*α*, IFN*γ*, and IL-2, while IL-5 and IL-4 release were not detected. The secretion of TNF*α*, IFN*γ*, and IL-2 is associated with a Th1 phenotype [41], which promotes an antitumor immune response [42].

**Figure 3:**
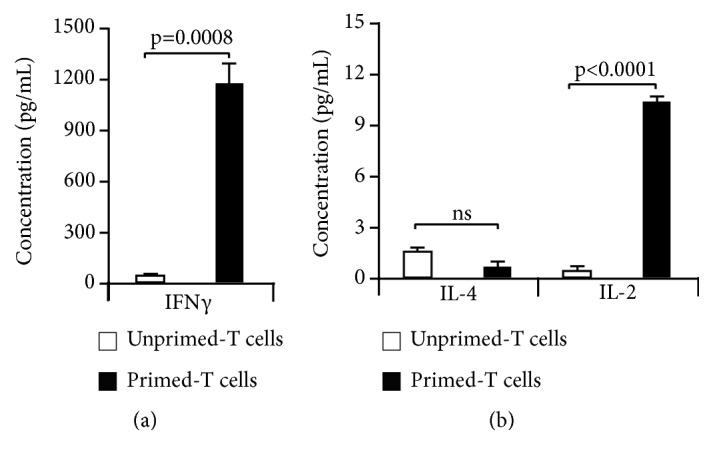
*IFN-γ and IL-2 secretion by unprimed or primed T cells cocultured with L5178Y-R cells.* (a) L5178Y-R cells were cocultured with unprimed T-lymphocytes (previously cocultured with unstimulated DCs-Control) or primed T-lymphocytes (previously cocultured with pulsed DCs-PKHB1-TCL) in a 1:5 tumor to effector ratio, for 24 h, and the supernatants were collected and assayed for (a) IFN-*γ* release by ELISA and (b) IL-4 and IL-2 release by FACS. Graphs represent the means (±SD) of three experiments performed independently.

Next, primed (cocultured with pulsed DCs-PKHB1-TCL) or unprimed (cocultured with unpulsed DCs) T-lymphocytes were collected and cocultured during 24 h with L5178Y-R cells (previously stained with calcein-AM). After 24 h of coculture, supernatants were obtained, and we assessed IFN*γ*, IL-4, and IL-2 cytokine release. A significant increase in IL-2 and IFN-*γ* release was observed in the supernatants of T-lymphocytes previously cocultured with DCs-PKHB1-TCL (Figure 3).

**Figure 4:**
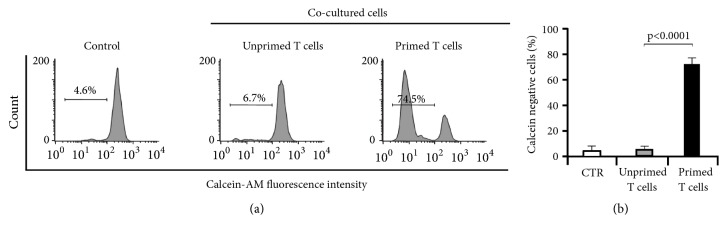
*Primed T cells' cytotoxicity in L5178Y-R cells.* (a) L5178Y-R cells were stained with calcein-AM and cocultured with unprimed T-lymphocytes (previously cocultured with unstimulated DCs-Control) or primed T-lymphocytes (previously cocultured with pulsed DCs-PKHB1-TCL) in a 1:5 tumor to effector ratio for 24 h. The percentage of L5178Y-R calcein negative cells was assessed by FACS; representative histograms are shown. (b) Graphs represent the means (±SD) of triplicates of three independent experiments obtained as in (a).

Once we observed that PKHB1-TCL induced IFN*γ* and IL-2 release, suggesting Th1 responses [41], we assessed antitumor cell cytotoxicity. For this purpose, we evaluated the loss of calcein in L5178Y-R cells. Results show that only T-lymphocytes cocultured with pulsed DCs-PKHB1-TCL induce a significant increase in the calcein negative L5178Y-R cells, in comparison with the T-lymphocytes cocultured with control DCs (not pulsed with PKHB1-TCL) (Figure 4). This confirms the correct antigen presentation by DCs-PKHB1-TCL and the T cell cytotoxicity against L5178Y-R cancer cells.

Detection of IL-2, INF*γ*, and TNF*α* in supernatants of DCs and T cell cocultures indicates the establishment of an efficient anticancer immune response. These observations are in agreement with the results observed in our cocultures of T cells with DCs-PKHB1-TCL. The secretion of these cytokines suggests a Th1 phenotype [41] which was confirmed by the loss in cell viability of L5178Y-R cells cocultured with primed T cells.

Several cytotoxic agents have been demonstrated to induce* ex vivo* antitumor T cell responses, such as bortezomib in myeloma [43] and doxorubicin in colon carcinoma [44]. Also the allogeneic off-the-shelf dendritic cell vaccine, currently in clinical trials for acute myeloid leukemia [45], has demonstrated these responses* ex vivo *and* in vivo *in patients.

### 3.4. PKHB1-TCL Induces Tumor Regression

**Figure 5:**
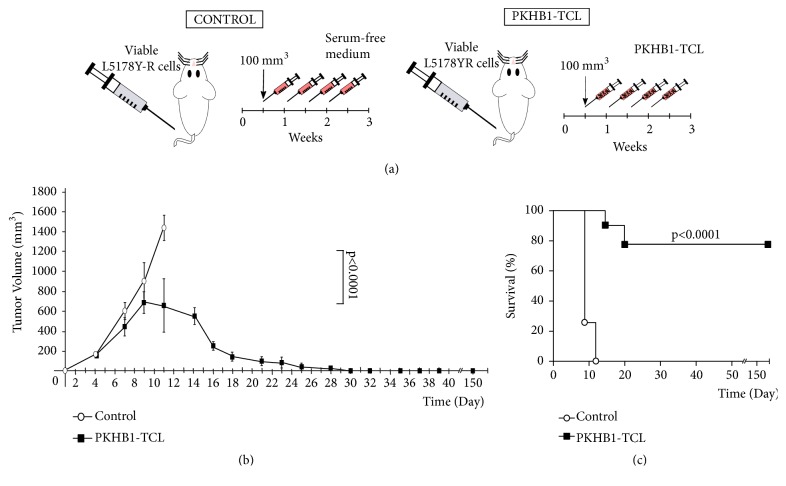
*PKHB1-TCL treatment in L5178Y-R-tumor-bearing mice induce tumor regression.* (a) Schema of PKHB1-TCL (5 × 10^6^ CC_100_ PKHB1-treated L5178Y-R cells) treatment started when tumor reached 100 mm^3^, and then PKHB1-TCL was administrated every 3 days for two weeks (for a total of four injections). (b) Graph indicates tumor volume (±SD) of untreated mice (control; n = 7) or PKHB1-TCL-treated mice (PKHB1-TCL; n = 9). (c) Kaplan-Meier survival curve of untreated mice (control; n = 7) or PKHB1-TCL-treated mice (PKHB1-TCL; n = 9) over time.

Once we established the* ex vivo* antitumor immune response induced by PKHB1-TCL, we assessed if the* in vivo* injection of PKHB1-TCL was able to diminish tumor growth and improve overall survival in syngeneic mice transplanted with L5178Y-R cells. First 2x10^6^ L5178Y-R cells were inoculated in BALB/c mice. When the tumor reached 100 mm^3^, a mice control-group was left without treatment (Control; n = 7), and a second group was treated with PKHB1-TCL two times per week (PKHB1-TCL; n = 9) (Figure 5(a)). Tumor growth was measured: we observed that PKHB1-TCL-treated mice showed significantly diminished tumor growth after day 10 (7 days after the first treatment), which continued to decrease until no tumor was detected by day 30 (Figure 5(b)). Tumor growth diminution was reflected in overall mice survival, as PKHB1-TCL-treated mice presented an 80% of survival over time (more than 150 days), while all control mice perished by day 11 (Figure 5(c)).

**Figure 6:**
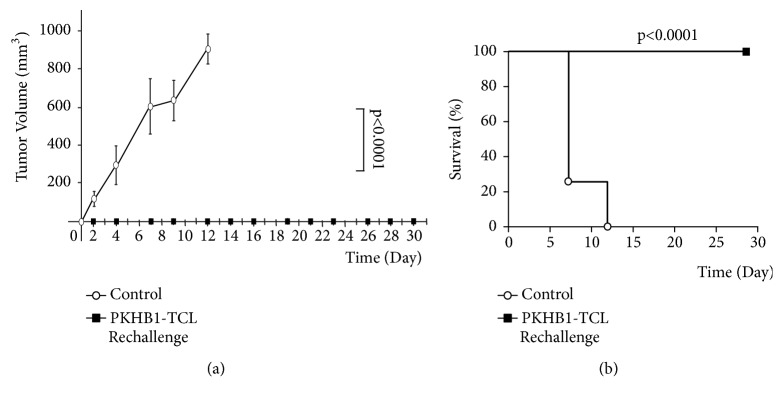
*PKHB1-TCL therapeutic vaccination induces long-term antitumor memory.* Mice in remission after therapeutic vaccinations were rechallenged with 2x10^6^ L5178Y-R viable cells. (a) Graph indicates tumor growth in control mice (control, n = 6) or mice in remission after a previous treatment with PKHB1-TCL that were rechallenged with living L5178Y-R cells (PKHB1-TCL-Rechallenge, n= 6). (b) Kaplan-Meier survival graph of mice treated as in (a) over time. Control: n = 6; PKHB1-TCL-Rechallenge, n= 6.

To assess immunological memory against tumor antigens after PKHB1-TCL-treatment, mice in complete remission (tumor free >60 days) were rechallenged with living L5178YR cells. Compared to naïve mice (control), in which a primary L5178YR cell challenge resulted in rapid tumor progression, those that were in remission were completely resistant to a rechallenge of L5178YR cells (Figure 6(a)). Furthermore, as no tumor developed, we observed a 100% of survival of the PKHB1-TCL-Rechallenged mice while all control mice perished by day 12 (Figure 6(b)).

It has been proved that other TCLs reduce tumor volume in different types of cancer, as, for example, combined with CpG in a glioblastoma mouse tumor model [46]. Additionally, in clinical trials TCLs have been tested in melanoma, prostate, mesothelioma, ovarian, and colorectal cancers [8]. These TCLs are usually produced using radiation, repeated freezing, and thawing, among others. Here we show that PKHB1-CD47 activation, which has been shown to effectively induce cell death in different types of cancer [21, 22], including cells coming from refractory patients [21, 22], can provide an immunogenic TCL which is able to promote an antitumor immune response, even in the absence of adjuvants.

Interestingly, we observed that tumor volume began to diminish 7 days after the first administration of PKHB1-TCL reaching tumor regression by day 28 (Figure 5(c)). This waiting time corresponds with the time needed for T cells to expand and activate an antitumor immune response [47].

Other types of TCL have been able to induce tumor regression, increasing the survival rate in patients with melanoma and prostate cancer [8]; however, they do so only in combination with adjuvants, such as the case of CpG oligodeoxynucleotides, which are TLR9-agonist [46].

We recently demonstrated that PKHB1 treatment of tumor-bearing mice induced long‐term tumor prevention in 90% of the mice that presented complete tumor regression [23]. Here we demonstrated that PKHB1-TCL induces long-term immunological memory against tumor antigens, preventing tumor establishment in 100% of the mice after L5178YR cells rechallenge (Figure 6). This underlines the immunogenicity of CD47-mediated cell death, when administering a CD47 agonist peptide or CD47-killed cells. This long lasting immunological memory has been promoted also by a TCL obtained by repeated freezing and thawing and radiation-treated glioma cells, where nearly a 100% of survival was observed [48].

Although immunotherapy with pulsed DCs, primed T-lymphocytes, or CAR-T cells is the principal approach used to stimulate antitumor immune responses, here we demonstrate that the crude TCL obtained from PKHB1 treatment is able to induce tumor regression in 80% of the mice, while preventing tumor establishment in 100% of the rechallenged mice that survived after PKHB1-TCL-treatment.

## 4. Conclusions

**Figure 7:**
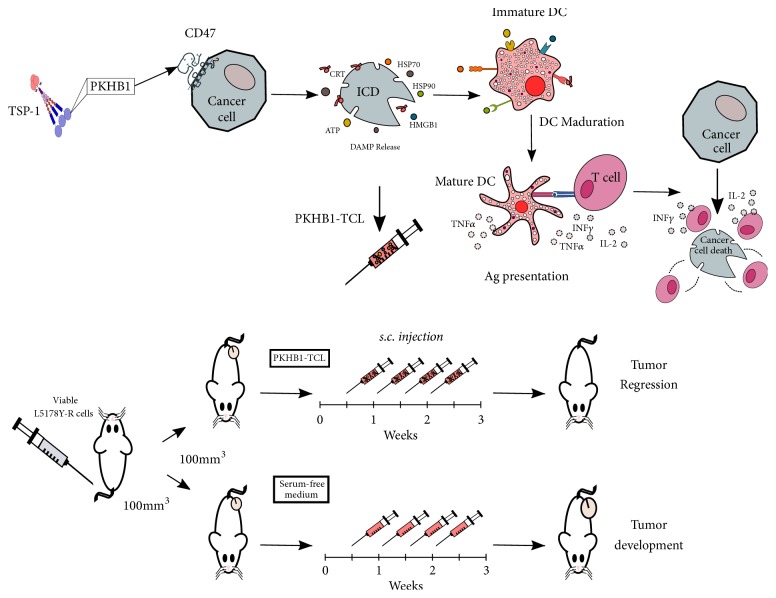
*Schematic representation of CD47-mediated immunogenic cell death in vitro, ex vivo, and in vivo.* PKHB1 induces selective ICD in L5178Y-R cell line leading to damage-associated molecular patterns (DAMP) release. DAMPs promote dendritic cell (DC) maturation and subsequence antigen presentation and T cell activation to induce cancer cell death. Moreover, PKHB1-treated cells administrated as a therapeutic vaccine induce tumor regression in syngeneic mice bearing L5178Y-R tumors. CRT, calreticulin; HMGB1, high-mobility group box 1; HSP, heat shock protein; ICD, immunogenic cell death; TSP-1, thrombospondin-1.

In this work we determined that the ICD induced by the CD47-agonist peptide, PKHB1, has a therapeutic potential, as the PKHB1-TCL was able to induce antitumor immune responses* ex vivo* and* in vivo *in an established L5178Y-R tumor. This was done by promoting the maturation of DCs, which trigger T cell antitumor effects, including INF*γ* release and L5178Y-R cell cytotoxicity, leading to tumor regression (Figure 7). Additionally, PKHB1-TCL-treated mice developed long-term immunological memory. These results highlight the immunogenicity of the cell death induced by CD47 activation by PKHB1 as a potential therapeutic tool to overcome the low immunogenicity of cancer cells, such as T-ALL.

## Figures and Tables

**Table 1:** TNF*α*, IFN*γ*, IL-5, IL-4, and IL-2 cytokine release (pg/mL) in cocultures of T lymphocytes with control or pulsed DCs.

	TNF*α* (pg/mL)	IFN*γ* (pg/mL)	IL-5 (pg/mL)	IL-4 (pg/mL)	IL-2 (pg/mL)
DCs-Control + T-lymphocytes	38.9 ± 14	0 ± 0	0 ± 0	0 ± 0	0.2 ± 0.2

DCs-PKHB1-TCL + T-lymphocytes	479.6*∗∗* ± 156	974.33*∗∗∗* ± 115	0 ± 0	0 ± 0	3.5*∗∗* ± 0.7

Bone marrow-derived murine DCs were left with medium alone (DCs-control) or pulsed (DCs-PKHB1-TCL) during 24h with a PKHB1-TCL. Then, DCs were cocultured during 4 days with T-lymphocytes, and the supernatants were collected to quantify TNF*α*, IFN*γ*, IL-5, IL-4, and IL-2 release, by FACS. Numbers represent the means (± SD) of triplicates of three independent experiments. *∗∗*p<0.01; *∗∗∗*p<0.001.

## Data Availability

The data used to support the findings of this study are available from the corresponding authors upon request.

## Supplementary Materials

Supplementary Materials
* Supplementary Table 1. TNFα, IFNγ, IL-5, IL-4, and IL-2 cytokine release by PKHB1-tumor cell lysate.* L5178Y-R cells were treated for 2 h with PKHB1 (300*μ*M) and the supernatants were collected to quantify TNF*α*, IFN*γ*, IL-5, IL-4, and IL-2 release, by FACS.
