# Supplementary material for: PKHB1 Tumor Cell Lysate Induces Antitumor Immune System Stimulation and Tumor Regression in Syngeneic Mice with Tumoral T Lymphoblasts
Source: J Oncol. 2019 Jun 4;2019:9852361. doi: 10.1155/2019/9852361 (PMC6582786; doi:10.1155/2019/9852361)
Supplement: Supplementary Materials — Supplementary Table 1. TNFα, IFNγ, IL-5, IL-4, and IL-2 cytokine release by PKHB1-tumor cell lysate. L5178Y-R cells were treated for 2 h with PKHB1 (300μM) and the supernatants were collected to quantify TNFα, IFNγ, IL-5, IL-4, and IL-2 release, by FACS. [file 9852361.f1.docx]

**Supplementary Material**

|  | TNFα | IFNγ | IL-5 | IL-4 | IL-2 |
| --- | --- | --- | --- | --- | --- |
| PKHB1-TCL | 1.47  ± 2.55 | 0.49  ± 0.42 | 0  ± 0 | 0.5  ± 0.55 | 1.075  ± 0.27 |

**Supplementary Table 1. TNFα, IFNγ, IL-5, IL-4, and IL-2 cytokine release by PKHB1-tumor cell lysate.** L5178Y-R cells were treated for 2h with PKHB1(300μM) and the supernatants were collected to quantify TNFα, IFNγ, IL-5, IL-4, and IL-2 release, by FACS.
